# Reduced Pain by Mind-Body Intervention Correlates with Improvement of Shoulder Function in People with Shoulder Pain: A Randomized Controlled Trial

**DOI:** 10.1155/2022/6149052

**Published:** 2022-03-24

**Authors:** Hojung Kang, Seung Chan An, Byeongkwan Kim, Youngjae Song, Jaesung Yoo, Eugene Koh, Seungheun Lee, Hyun-Jeong Yang

**Affiliations:** ^1^Korea Institute of Brain Science, Seoul 06022, Republic of Korea; ^2^Bomunsan Hospital, Daejeon 35052, Republic of Korea; ^3^Temasek Life Sciences Laboratories, Singapore 117604, Singapore; ^4^Department of Integrative Health Care, University of Brain Education, Cheonan, Republic of Korea

## Abstract

Meditation and acupressure-like stimulations have been shown to relieve pain. The aim of this study was to determine whether a short bout of mind-body intervention combined with meditation and acupressure-like stimulation was able to alleviate shoulder pain and improve its function in a short time window. Sixty-five adults with shoulder pain were recruited and randomly classified into two groups. One group participated in an intervention which consisted of acupressure-like stimulation and meditation over a 5 min period. The other group was instructed to rest during this time. A visual analog scale (VAS) pain score and objective constant scores were measured before and after intervention to determine shoulder pain and range of motion (ROM), respectively. A two-way repeated measures analysis of variance with Bonferroni correction and a regression analysis were performed. VAS pain, objective constant score, flexion, abduction, and external rotation score showed significant interactions between time and group. The pain intensity was significantly reduced, while flexion and abduction were significantly improved, in the experimental group compared to the control group, after the intervention. In addition, the change of flexion negatively correlated with the change of pain intensity in the experimental group, but not in the control group. These results show that a short-term application of mind-body intervention significantly alleviates shoulder pain and improves shoulder movement, suggesting its potential use as a therapy for people with shoulder pain.

## 1. Introduction

Shoulder pain is one of the most common musculoskeletal complaints. A significant percentage of patients seeking medical attention have shoulder pain. The reported lifetime prevalence of shoulder pain ranges from 6.7% to 66.7% of the population [1]. The shoulder is a complex joint whose function is to position the hand for activities of daily living, work, and sports [2]. Failure of this mechanism can have a dramatic effect on one's lifestyle. A large number of patients with shoulder pain are treated nonoperatively by using alternative interventions such as massage, physiotherapy, yoga, and meditation [3–6].

Meditation shows promise for alleviating chronic pain [7, 8]. In a recent meta-analysis of 30 RCTs, meditation was related with a small decrease of pain compared to different types of controls [9]. Reduction of pain by meditation is associated with activation of the following brain areas: subgenual ACC for cognitive and affective pain control, orbitofrontal cortex for supporting contextual evaluation of sensory events, and right anterior insula for afferent nociceptive signal modulation and interoception awareness [10, 11]. Moreover, ascending nociceptive signals in the thalamus were also downregulated by executive attention during meditation [10, 11]. A series of changes in the brain induced by meditation suggests that meditation reduces pain through recontextualizing the pain as innocuous sensory information. The pain modulatory pathway based on mindfulness is mediated nonopioidergically [12], distinct from the placebo-based pain relief pathway [11, 12]. In a recent study, intravenous administration of an opioid antagonist naloxone does not antagonize meditation-induced pain relief, suggesting that pain relief by meditation is possibly mediated by the nonopioid pathway [13]. Moreover, meditation techniques often include breathing modulation, and in a study which investigated the role of breathing on pain relief, slow-paced breathing was suggested to relieve pain via the nonopioid pathway [14], showing that the breathing component in meditation is an effective means of pain regulation.

Acupressure has been studied extensively as a method for pain management. A meta-analysis of 15 studies showed that acupressure is effective for relieving a variety of pains including dysmenorrhea, labor pain, low back pain, chronic headache, and other traumatic pain [15]. In a recent systemic review about the effects of auricular acupressure on pain management, 12 studies showed a significant improvement in the pain outcomes of auricular acupressure compared with the control groups [16]. In a randomized controlled trial (RCT) of 33 women suffering from chronic neck pain, acupressure provided significant pain relief [17]. In another RCT with 24 individuals with chronic neck pain, a manual therapy technique reduced the visual analog scale (VAS) pain score and increased range of motion (ROM) of the neck [18]. In an RCT with 62 patients who had undergone a total knee replacement operation, a 3-day postoperation auricular acupressure treatment significantly reduced the use of analgesic drug usage, indicating relief of postoperative pain by acupressure and significantly improved passive knee motion on the 3rd day after surgery [19]. When pain is reduced by various methods as above, improvements in ROM of joints are also observed. In professional tennis players, shoulder ROM seems to be associated with shoulder pain history [20], supporting the association between pain and motion.

As described above, mind-body interventions such as meditation and acupressure have the potential to relieve pain intensity and improve joint mobility. In this study, we performed a randomized controlled trial for people with shoulder pain under the hypothesis that a short-term intervention which combined acupressure-like stimulation and meditation may help to relieve shoulder pain and improve shoulder function.

## 2. Materials and Methods

### 2.1. Participants

**Figure 1:**
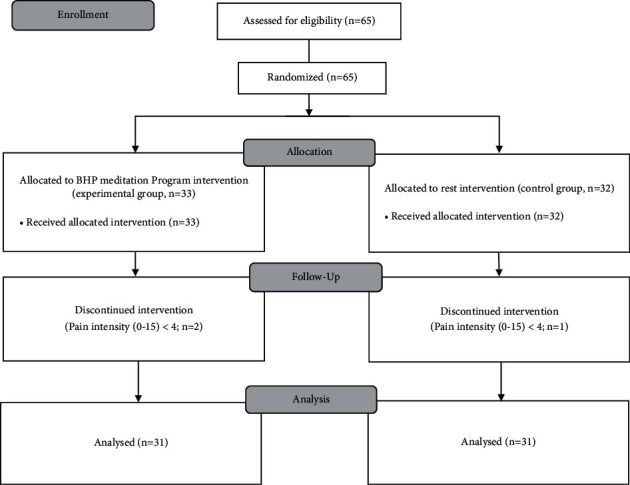
Constant 2010 flow diagram. Sixty-five participants were recruited. The participants were randomly divided into two groups: experimental and control groups. Sixty-two participants (experimental, 31; control, 31) completed the study, with 3 dropouts (experimental, 2; control, 1). The dropouts showed less than 4 points of pain intensity.

The participants were recruited through flyers and online and offline poster announcements (local clinics and public centers). The inclusion criteria were as follows: people with shoulder pain for at least 2 months, people who consented to participate in the research voluntarily, and people who could hear the explanation of the experiment (are not hearing impaired), read, understand the manual, speak their opinions, and follow the instructions. Exclusion criteria were as follows: people with shoulder pain due to rheumatic arthritis, osteoarthritis, bone defect injury, osteoporosis, and malignant tumor and people with low pain intensity (VAS pain score <4) during shoulder movement of flexion, abduction, internal rotation, and external rotation. Based on a pilot study, a sample size of 27 participants per group was sufficient to give over 90% of power with an alpha level set at 0.05 for a two-tailed unpaired *t*-test model (g-power software 3.1.9.7). Considering the dropout rate, a total of 65 participants were recruited. Sixty-five people volunteered for the study and were randomly divided into two groups: the experimental (*N* = 33) and control (*N* = 32) groups. Two and one individuals were dropped from each group, respectively, due to low pain intensity (VAS pain score <4) during ROM measurement (Figure 1).

All participants were given an explanation of the experiments and participated in the measurement of four subscales (pain, activities of daily living, ROM (flexion, abduction, internal rotation, and external rotation), and strength) of the constant score and VAS pain intensity. Constant score has been adopted as an official tool for assessing the shoulder by the European Society for Surgery of the Shoulder and the Elbow [21]. The intervention protocol was applied for 5 min for individuals in the experimental group, while the participants of the control group took rest. After the intervention period, VAS pain intensity and two subscales (ROM and strength) of the constant score were measured again. To minimize bias, the participants were asked not to reveal information to the evaluators about the treatment to which they had been assigned. Participants were rewarded with 50,000 KRW for their participation. The research has been carried out in accordance with The Code of Ethics of the World Medical Association (Declaration of Helsinki) for experiments involving humans. All subjects signed an informed consent form before their inclusion. The Institutional Review Board of the University of Brain Education approved this study. All the experiments were performed in Bomunsan Hospital in Daejeon, Korea. The current protocol is registered as a clinical trial in the Clinical Research Information Service (CRIS registration number: KCT0005602).

### 2.2. BHP Meditation Intervention

Brain education meditation (BEM) (also referred as brain wave vibration meditation) is a modernized mind-body training method which is rooted in Korean Sundo tradition and consists of several techniques including exercises which improves the connection between the brain and body, such as qigong, breathing postures, body awareness meditation, and brain wave vibration meditation with rhythmic body movement [22–28], and has been investigated for its differences from other mind-body interventions such as mindfulness meditation and yoga [23, 26]. BEM is associated with its effects on various body systems such as changes in brain structure and function [24, 29–31], improvements in emotion and cognition [32–34], and suppression of inflammation [25, 35]. Brain education healing point (BHP) meditation, which is used in the current study, is a meditation program that combines acupressure-like stimulation with the meditation of the abovementioned BEM tradition [36]. During BHP meditation, specific body points are stimulated with pressure, and improvements of the body area of attention are visualised via imagery meditation combined with breathing regulation [36]. BHP meditation is a short and intense meditation program that can lead even beginners to deep meditation.

BHP meditation consists of acupressure-like stimulation and breathing/body awareness meditation. The first step in BHP meditation is to find a “healing point,” where subjects report more pain than other places when a specific part of the body is pressed. This point is called the BHP point and can be found anywhere on the body. BHP meditation first applies acupressure-like stimulation and then induces relaxed attention by body awareness and imagery meditation combined with breathing [36]. In this study, we restricted the BHP point to within 0.5 cm from the end of the eponychium to exclude confounding factors related to body parts. This specific region is most frequently used for BHP meditation [36]. The second step is to press the BHP point with moderate force using a BHP finder (HSP World, AZ), which resembles a thick pen with a blunt end. The BHP points of all participants were stimulated by one experienced trainer to remove variability. The trainer pressed the BHP point with moderate force for few seconds, released, and repeated this cycle for 1 min. After 1 min of pressing the BHP point, participants were guided to meditate on body awareness with breathing for an additional 4 min under the trainer's guidance. The total participation time for BHP meditation was 5 min.

### 2.3. Control Condition

A waitlist control group design was employed. Participants in the control group were seated and asked to relax for 5 min. Data were collected from the control group on the same schedule as the experimental group. Control participants were offered the BHP mediation program only after the experiments. No further data were collected from control participants at this time point.

### 2.4. Constant Score

The Constant score was divided into four subscales, including pain during the last 24 h (15 points maximum), activities of daily living for the last one week (20 points maximum), range of motion (ROM; 40 points maximum), and strength (25 points maximum) [21]. For ROM, abduction, flexion, internal rotation, and external rotation were measured. To exclude any bias during measurement, physical therapists were blinded from the details of the experiments and technically measured ROM and pain score. Strength was tested with scoring based on the number of kilograms of pull the patient can resist in abduction for 3 seconds, up to a maximum of 90°. The higher the score, the better the quality of function (minimum 0, maximum 100). The subjective scores (i.e., pain for last 24 h and activities of daily living for the last 1 week) were used for demographics, and the objective scores (i.e., ROM and strength before and after the intervention) were compared to analyze shoulder function. The combination of subjective and objective tests to estimate shoulder function is given in Supplementary Table 1. Additionally, the pain intensity experienced by the participant during ROM activities was measured on a 16-point numerical pain VAS.

### 2.5. Statistical Analysis

The association between BHP meditation and shoulder pain was analyzed using two-way repeated analysis of variance (ANOVA), including the between-subjects factor of intervention condition (BHP meditation or control) and the within-subjects factor of assessment time. For the constant scores, the assessments included pretreatment scores (before the BHP meditation session) and posttreatment scores (after the BHP meditation session). Multiple comparisons were corrected using critical values from the *t* distribution after Bonferroni adjustment. Regression analysis was performed to confirm the relationship between the change of pain intensity and the change of each objective constant score affected by the intervention.

## 3. Results

### 3.1. Demographics

A total of 65 subjects participated in the current study. The distribution of gender was similar in both groups (*p*=0.8041, *χ*^2^ test, Table 1). The age of the experimental group (57.48 ± 10.33 years old) and control group (59.28 ± 11.82 years old) did not differ significantly (*p*=0.5161, Student's *t*-test). The subjective subscales of constant score, including sleep, work, recreation, position, and pain scores, were not significantly different between the two groups, as given in Table 1.

### 3.2. Constant Score and Pain Intensity

**Figure 2:**
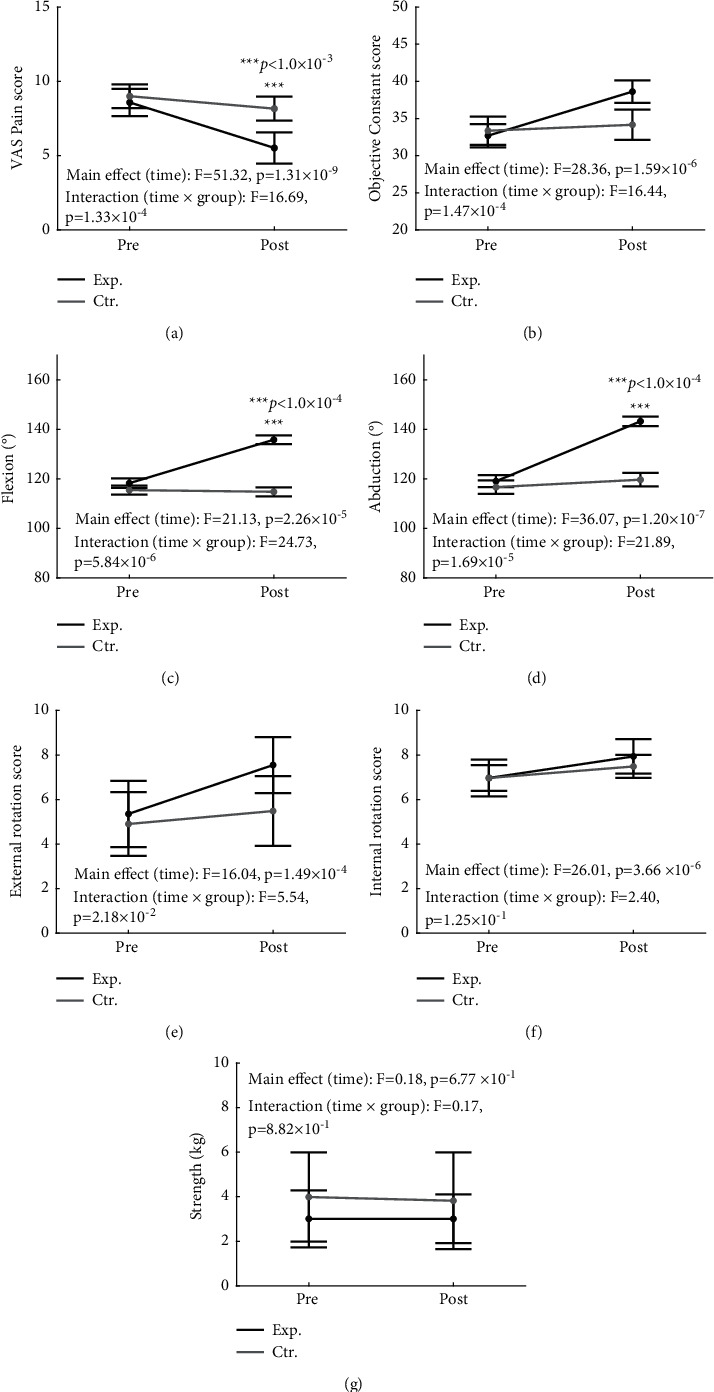
The effects of the intervention on pain, range of shoulder movement, and shoulder strength. Two-way repeated measures ANOVA of following measurements are indicated: (a) VAS pain score; (b) objective constant score; (c) flexion; (d) abduction; (e) external rotation score; (f) internal rotation score; (g) strength. Post hoc Bonferroni correction, *p* < 1.0 × 10^−3^, ^*∗∗∗*^. The dots and error bars of pre and postintervention indicate mean ± SD.

The VAS pain score was analyzed via a two-way repeated measures ANOVA with group (experimental/control) and time (pre/post) as factors. Pre/postmeasurement results in experimental and control groups are given in Supplementary Table 2. We found a main effect in the time factor (*F* (1, 60) = 51.32, *p*=1.31 × 10^−9^, *η*_*p*_^2^ = 0.461, Figure 2(a)). In the interaction analysis between the group and time factors, a significant interaction was found (*F* (1, 60) = 16.69, *p*=1.33 × 10^−4^, *η*_*p*_^2^ = 0.218, Figure 2(a)). Post hoc tests using the Bonferroni correction revealed that there was no significant difference between the experimental group and the control group before intervention in the VAS pain score. However, the VAS pain score of the experimental group was significantly lower compared with that of the control group after the intervention (experimental group = 5.52 ± 1.05, control group = 8.16 ± 0.81, *p*=3.76 × 10^−4^, Figure 2(a)). For the objective constant score, there was a main effect in the time factor (*F* (1, 60) = 28.36, *p*=1.59 × 10^−6^, *η*_*p*_^2^ = 0.321, Figure 2(b)) and interaction between the group and time factors (*F* (1, 60) = 16.44, *p*=1.47 × 10^−4^, *η*_*p*_^2^ = 0.215, Figure 2(b)). However, no significant difference was found in the group factor. For the flexion, abduction, and external rotation score, significant results were found in the main effect of time (*F* (1, 60) = 21.13, *p*=2.26 × 10^−5^, *η*_*p*_^2^ = 0.26, Figure 2(c); *F* (1, 60) = 36.07, *p*=1.20 × 10^−7^, *η*_*p*_^2^ = 0.38, Figure 2(d); *F* (1,60) = 16.04, *p*=1.49 × 10^−4^, *η*_*p*_^2^ = 0.21, Figure 2(e), respectively) and the interaction between group and time factors (*F* (1, 60) = 24.73, *p*=5.84 × 10^−6^, *η*_*p*_^2^ = 0.29, Figure 2(c); *F* (1, 60) = 21.89, *p*=1.69 × 10^−5^, *η*_*p*_^2^ = 0.27, Figure 2(d); *F* (1,60) = 5.54, *p*=2.18 × 10^−2^, *η*_*p*_^2^ = 0.08, Figure 2(e), respectively). In internal rotation, there was a significant main effect of time; however, no significant interaction between group and time factors was found (Figure 2(f)). In strength, there were no significant main effects of time (Figure 2(g)). Post hoc tests using the Bonferroni correction revealed that there was no significant difference between the experimental group and the control group before the intervention in the flexion and abduction performance. However, after the intervention, it was confirmed that the flexion and abduction performance of the experimental group was significantly improved compared to the control group (experimental group = 136 ± 2°, control group = 115 ± 2°, *p*=7.27 × 10^−5^; experimental group = 143 ± 2°, control group = 120 ± 3°, *p*=6.36 × 10^−4^, respectively, with Bonferroni post hoc test).

**Figure 3:**
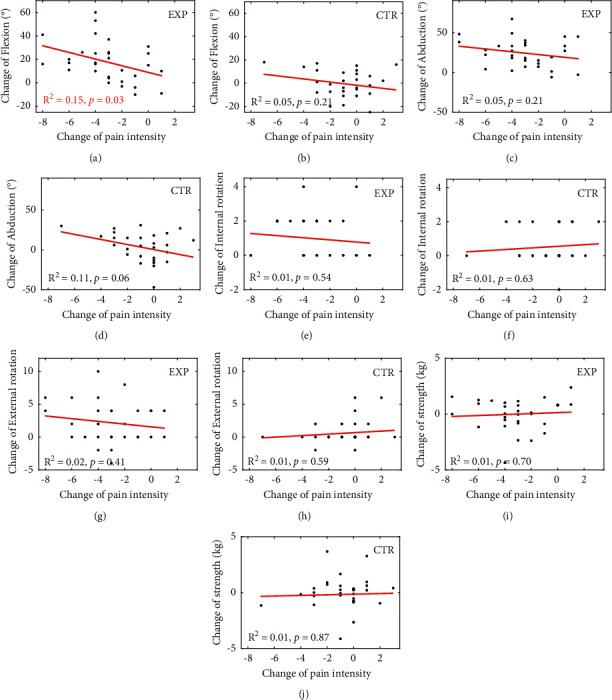
Regression analysis between change of each objective task of constant score and change of VAS pain score. Regression analysis of change of VAS pain score with change of flexion ((a)-(b)), abduction ((c)-(d)), internal rotation ((e)-(f)), external rotation ((g)-(h)), and abduction strength ((i)-(j)). Results of experimental ((a), (c), (e), (g), (i)) and control ((b), (d), (f), (h), (j)) groups are indicated. EXP, experimental group. CTR, control group.

According to the regression analysis for confirming the relationship between the change of pain intensity and the change of each objective constant score (Figure 3), VAS pain score change was associated with a change in forward flexion (*R*^2^ = 0.15, *p*=0.03) in the experimental group (Figure 3(a)) but not in the control group (Figure 3(b)). The obtained *R*^2^ value for endogenous variables is greater than 0.1, which is deemed adequate [37]. No other objective constant score task changes were significantly associated with pain intensity in either group (Figures 3(c)–3(j)).

Our results indicated that BHP meditation intervention significantly reduced shoulder pain during motion (Figure 2(a)) and improved shoulder ROM (Figures 2(c)–2(d)). Moreover, the pain reduction was associated with the improved forward flexion (Figure 3(a)), one of the tasks for shoulder function evaluation.

## 4. Discussion

In this study, we aimed to examine the effects of mind-body intervention on the treatment and recovery of shoulder pain. Previous work has shown the effects of mind-body intervention on pain relief [9, 38] and the relationship between pain and motion of joints [39, 40]. Acupressure techniques have been reported to reduce pain and improve joint maneuverability [15, 18, 19, 41]. BEM has also been studied for its effects on inflammation reduction [25, 35], which exacerbate pain [42, 43]. In this study, we found that BHP intervention, which combines acupressure-like stimulation with BEM, can help relieve pain and improve shoulder motion for people with chronic shoulder pain.

The beneficial effects of meditation on pain reduction have been reported [44–46]. As meditation contributes to pain reduction via the reinterpretation of the nociceptive signal [47], the meditative component of BHP intervention may contribute to pain reduction via such mechanisms. Additionally, different types of breathing training such as virtual reality breathing and traditional mindful breathing also appear to improve pain thresholds. Interestingly, stimulation of different brain regions has been reported which varies depending on the breathing training method [48]. This suggests that breathing may have also independently affected pain regulation.

The acupressure-like component of BHP intervention may affect pain signaling which can be explained by gate control theory. During BHP intervention, pressure is applied to the participant's fingertips [36]. In the gate control theory of pain reduction, Melzack and Wall theorized that the experience of pain can be reduced by competing stimuli, such as pressure or cold, because these stimuli travel along faster nervous system pathways than pain [49]. In this way, stimulating the fingertips with sufficient pressure may interfere with the transmission of preexisting chronic shoulder pain to the brain, effectively “closing the gate” to the reception of pain before it can be processed.

Among the shoulder motions comprising the objective constant scores (i.e., flexion, abduction, internal rotation, and external rotation), BHP affected flexion and abduction, but not internal and external rotation. Each motion uses the following muscles: anterior deltoid, pectoralis major, and coracobrachialis for flexion [50]; supraspinatus, deltoid, trapezius, and serratus anterior for abduction [51]; subscapularis, latissimus dorsi, teres major, and deltoid (anterior fiber) for internal rotation [52]; and infraspinatus and teres minor for external rotation [52]. Therefore, the effect of BHP may be associated with the supraspinatus, trapezius, serratus anterior, pectoralis major, and coracobrachialis muscles which are related with flexion and abduction, although more measurements such as electromyogram data are required to make more concrete conclusions.

Furthermore, we found that pain intensity was negatively correlated with flexion in the BHP intervention group (Figure 3(a)). Previous work has also shown the relationships between pain intensity and shoulder function. During a repetitive shoulder flexion task, upper trapezius muscle pain induced reorganization in the coordinated activity of the subdivisions of the trapezius muscle [39]. In a study which investigated the contributing factors for shoulder function among 142 subjects with nonoperative shoulder disorders, pain intensity was found to contribute significantly to shoulder function score [40]. Based on the previous reports between pain reduction and performance improvement, it is plausible to think that the improved flexion performance may be at least partially contributed by pain reduction via BHP intervention. Compared to this, abduction performance was significantly improved regardless of pain intensity after BHP (Figure 2(d)). Abduction performance may respond to pain reduction with high sensitivity, i.e., exhibiting significant performance improvement under even a small pain reduction, although the mechanism requires further study.

The short nature of the intervention in this study currently only provides insights into the short-term effects of BHP intervention. In this study, the single short-term BHP intervention contributes to shoulder pain reduction and improvement in function. We surmise that the observed effects are likely to be direct responses of BHP intervention as they occurred within a short time window (i.e., 5 minutes). However, it is not known if BHP intervention over a longer-term period would retain its effectiveness as a treatment for shoulder pain; thus, data on the effectiveness of longer-term BHP interventions remains to be examined in a future study.

The method of recruiting participants is an additional limitation in this study. The participants applied for care on their own initiative and, thus, may have a positive attitude toward the treatment. This positive attitude may have created a group with high expectations. In addition, the control group did not receive a placebo. To minimize bias, the patients were asked not to reveal information to the evaluators about the treatment to which they had been assigned. We used an intention-to-treat analysis, provided standardized information to the participants, and used reliable and valid outcome measures. However, as the control group was a rest control rather than an active control or a placebo group, we cannot confirm if the effects of the intervention would be better than the placebo or other preexisting methods.

## 5. Conclusions

This RCT revealed that a single short-term BHP meditation not only relieves pain but also improved ROM (i.e., flexion and ablation) performance. Interestingly, flexion performance was found to be significantly related to pain reduction. These results indicate that pain reduction through BHP meditation has a direct effect on improving shoulder range of motion. Further studies utilizing active controls or comparisons against other therapies would provide more insight into the efficacy of BHP as a therapy for shoulder pain. Also, studies of long-term BHP intervention could validate that this intervention is effective in prolonging the effects of pain reduction and shoulder mobility observed here. The reduction of shoulder pain and functional improvements by BHP intervention would significantly contribute to improving the quality of life for patients with chronic shoulder pain.

## Figures and Tables

**Table 1:** Demographics.

Characteristics	Experimental group (*n* = 33)	Control group (*n* = 32)	Statistics	
			*χ* ^2^ or *t*	*P*
Gender, *n* (male/female)	7/26	6/26	0.0616	0.8041
Ages, years	57.48 ± 10.33	59.28 ± 11.82	0.6530	0.5161
Sleep	0.85 ± 0.67	1.06 ± 0.67	1.2912	0.2013
Work	2.09 ± 0.98	2.09 ± 1.15	0.0108	0.9915
Recreation	2.09 ± 1.04	1.71 ± 0.89	1.5475	0.1268
Position	6.06 ± 2.85	6.19 ± 2.40	0.1938	0.8469
Pain	6.24 ± 3.21	6.59 ± 3.22	0.4403	0.6613

Values indicate mean ± SD. Scores of sleep, work, recreation, and position, which are subjective subscales of constant score, were assessed based on the previous week, while pain score was obtained by assessment based on the previous 24 h.

## Data Availability

The data generated or analyzed during this study are included within the article and Supplementary Materials.

## Supplementary Materials

Supplementary Materials Supplementary Table 1. The constant scoring system for individual parameters. Supplementary Table 2. Pre/postmeasurement results in experimental and control groups.
