# Supplementary material for: Reduced Pain by Mind-Body Intervention Correlates with Improvement of Shoulder Function in People with Shoulder Pain: A Randomized Controlled Trial
Source: Evid Based Complement Alternat Med. 2022 Mar 24;2022:6149052. doi: 10.1155/2022/6149052 (PMC8970874; doi:10.1155/2022/6149052)
Supplement: Supplementary Materials — Supplementary Table 1. The constant scoring system for individual parameters. Supplementary Table 2. Pre/postmeasurement results in experimental and control groups. [file 6149052.f1.docx]

## Supplementary Materials

**Supplementary Table 1. Constant scoring system for individual parameters.**

| **Points** | | | |
| --- | --- | --- | --- |
| **Pain (for last 24 hr)** | | |  |
| **None** | | | **15** |
| **Mild** | | | **10** |
| **Moderate** | | | **5** |
| **Severe** | | | **0** |
| **Activities of daily living (for last 1 week)** | | |  |
| **Activity level** | | |  |
| **Full work** | | **4** | |
| **Full recreation/sport** | | **4** | |
| **Unaffected sleep** | | **2** | |
| **Positioning** | | |  |
| **Up to waist** | | | **2** |
| **Up to xiphoid** | | | **4** |
| **Up to neck** | | | **6** |
| **Up to top of head** | | | **8** |
| **Above head** | | | **10** |
| **Total** | | | **20** |
| **Range of Motion** | | |  |
| **Flexion** | | | **10** |
| **Abduction** | | | **10** |
| **External rotation** | | | **10** |
| **Internal rotation** | | | **10** |
| **Power (1 point per pound of weight held in abduction by arm at 90°)** | | | **25** |
| **Total** | | | **100** |

**Supplementary Table 2. Pre/Post measurement results in experimental and control group.**

| **Group** | **Time** | **VAS pain score** | **Objective Constant score** | **Flexion (°)** | **Abduction (°)** | **Internal rotation score** | **External rotation score** | **Strength (kg)** |
| --- | --- | --- | --- | --- | --- | --- | --- | --- |
| **EXP** | **Pre** | 8.58±0.92 | 32.69±1.56 | 118±2 | 119±2 | 5.35±1.49 | 6.97±0.83 | 3.01±1.28 |
|  | **Post** | 5.52±1.05 | 38.62±1.52 | 136±2 | 143±2 | 7.55±1.26 | 7.94±0.77 | 3.01±1.09 |
| **CTR** | **Pre** | 9.00±0.79 | 33.35±1.89 | 115±2 | 117±3 | 4.90±1.43 | 6.97±0.58 | 3.99±2.01 |
|  | **Post** | 8.16±0.81 | 34.16±2.03 | 115±2 | 120±3 | 5.48±1.56 | 7.48±0.52 | 3.82±2.17 |

Values indicate mean ± SD.
